# HER3 genomic gain and sensitivity to gefitinib in advanced non-small-cell lung cancer patients

**DOI:** 10.1038/sj.bjc.6602865

**Published:** 2005-11-15

**Authors:** F Cappuzzo, L Toschi, I Domenichini, S Bartolini, G L Ceresoli, E Rossi, V Ludovini, A Cancellieri, E Magrini, L Bemis, W A Franklin, L Crino, P A Bunn, F R Hirsch, M Varella-Garcia

**Affiliations:** 1 Department of Medicine/Medical Oncology and Pathology, University of Colorado Cancer Center, Campus Box 8117; PO Box 6511, Aurora, CO 80045, USA; 2 Department of Medical Oncology, Bellaria-Maggiore Hospital, Bologna, Italy; 3 CINECA-Interuniversity Consortium, Bologna, Italy; 4 Department of Medical Oncology, Scientific Institute University Hospital San Raffaele, Milano, Italy; 5 Department of Medical Oncology, Policlinico Monteluce, Perugia, Italy

**Keywords:** HER3, EGFR, tyrosine kinase inhibitor, gefitinib, non-small-cell lung cancer

## Abstract

In non-small-cell lung cancer (NSCLC), sensitivity to tyrosine kinase inhibitors (TKIs) is associated with activating mutations and genomic gain of the epidermal growth factor receptor (EGFR). Preclinical data suggested that HER3 overexpression increases sensitivity to TKIs. A total of 82 NSCLC patients treated with gefitinib (250 mg), and previously evaluated for EGFR and HER2 status by fluorescence *in situ* hybridisation (FISH) and DNA sequencing, and for Phospho-Akt status by immunohistochemistry, were investigated for HER3 genomic gain by FISH. Patients with high polysomy and gene amplification were considered as HER3 FISH positive (+). HER3 FISH+ pattern was significantly associated with female gender (*P*=0.02) and never smoking history (*P*=0.02). Patients with HER3+ tumours (26.8%) had a significantly longer time to progression (3.7 *vs* 2.7, *P*=0.04) than patients with HER3− tumours, but not a significantly better response rate or survival. Patients with EGFR+/HER3+ tumours had higher objective response rate (36.4 *vs* 9.9%, *P*=0.03) and time to progression (7.7 *vs* 2.7 months, *P*=0.03) than patients with EGFR− and/or HER3− tumours, but no significantly longer survival. No difference in response was observed according to HER3 status in patients with EGFR+ tumours. Patients with HER2+/HER3+ tumours had similar outcome as patients with HER2− and/or HER3− tumours. Significantly different clinical end points were not observed between patients with HER3+/P-Akt+ and HER3− and/or P-Akt− tumours. Genomic gain for HER3 is not a marker for response or resistance to TKI therapy in advanced NSCLC patients.

Non-small-cell lung cancer (NSCLC) has been the leading cause of cancer death in the world (Greenlee *et al*, 2001). Platinum-based chemotherapy is the standard of care for advanced NSCLC, but even with newly developed chemotherapy strategies the median survival rarely exceeds 9 months and the fraction of patients alive after 1 year is approximately 30% (Non-small cell Lung Cancer Collaborative Group, 1995; Kelly *et al*, 2001; Schiller *et al*, 2002). In recent years, several new agents able to interfere with critical oncogenic mechanisms have been evaluated in clinical trials. Among them, the most promising classes of compounds are those targeting tyrosine kinases. The epidermal growth factor receptor (EGFR), including EGFR (Erb-B1), HER2/neu (Erb-B2), HER3 (Erb-B3) and HER4 (Erb-B4), is a family of receptor tyrosine kinases representing ideal therapeutic targets because they play a critical role in cancer proliferation and survival (Woodburn, 1999). A number of different ligands, including EGF, transforming growth factor alpha and neuregulins, activate these receptors by binding to the extracellular domain and inducing the formation of receptor homodimers or heterodimers followed by internalisation of the receptor/ligand complex and autophosphorylation. As a result, the tyrosine kinase signal transduction pathways are activated (Carpenter and Cohen, 1979; Yarden and Ullrich, 1988).

During the last decade, several new agents interfering with the EGFR family activity have been developed (Ciardiello *et al*, 2003). Among the most promising of these new drugs are gefitinib (ZD 1839, Iressa®, AstraZeneca, UK) and erlotinib (OSI 774, Tarceva®, Genentech, USA). Both are orally active, selective EGFR tyrosine kinase inhibitors (EGFR-TKI) that demonstrated anti-tumoural activity in approximately 10% of unselected NSCLC (Fukuoka *et al*, 2003; Kris *et al*, 2003), with a survival benefit when compared with placebo that was statistically significant only in the case of erlotinib (Shepherd *et al*, 2005; Thatcher *et al*, 2005). Molecular mechanisms underlying TKI sensitivity have been recently discovered. Patients with specific EGFR gene mutations and/or genomic gain demonstrated to be particularly sensitive to the inhibitory effects of TKIs (Lynch *et al*, 2004; Paez *et al*, 2004; Pao *et al*, 2004; Cappuzzo *et al*, 2005a; Hirsch *et al*, 2005; Tsao *et al*, 2005), although a favourable prognostic role of EGFR gene mutations cannot be ruled out (Bell *et al*, 2005; Eberhard *et al*, 2005). It is also known that EGFR mutations activated the antiapoptotic protein Akt (Sordella *et al*, 2004), and that patients whose tumours have activated Akt are more sensitive to TKIs than patient whose tumours are phospho-AKT negative (Cappuzzo *et al*, 2004).

Among the four members of the EGFR family, HER3 is unique because of its catalytically deficient kinase domain (Guy *et al*, 1994) and its high propensity to self-associate in the absence of ligand (Landgraf and Eisenberg, 2000), and the ability to assume a locked conformation, using an intramolecular tether (Cho and Leahy, 2002). HER3 signalling relies on heterodimerisation with other EGFR family member, preferentially HER2 (Sliwkowski *et al*, 1994). Simultaneous overexpression of both HER2 and HER3 was found in several cancers (Naidu *et al*, 1998; Krahn *et al*, 2001), and increased drug resistance in many HER2-overexpressing cancers depends on increased levels of HER3 or EGFR (Chen *et al*, 2000).

Because of the rich crosstalk among the EGFR family members, the TKI sensitivity is not only directly dependent on the presence of EGFR but also largely influenced by other family members, particularly HER2. In previous studies, we observed that HER2 gene gain (Cappuzzo *et al*, 2005b) but not HER2 protein expression (Cappuzzo *et al*, 2003) was related to gefitinib sensitivity, and EGFR+ patients with increased HER2 gene copy number had a significantly higher response rate, time to progression and survival. Moreover, preclinical data indicated that gefitinib inhibits cell proliferation by sequestration of HER2 and HER3 receptors in an inactive heterodimer configuration with EGFR (Anido *et al*, 2003).

In previous studies, we also reported that activation of the antiapoptotic protein Akt was significantly associated with gefitinib sensitivity (Cappuzzo *et al*, 2004), with a significant improvement in survival only when Akt activation was sustained by an EGFR-dependent mechanism (Cappuzzo *et al*, 2005a). Moreover, it is known that EGFR-mediated activation of Akt requires the activation of phosphatidylinositol 3 kinase (PI3K), and this can occur via dimerisation of EGFR with HER3, which is able to directly couple to PI3K (Fedi *et al*, 1994). Finally, a recent study showed that HER3 is used to couple EGFR to the PI3K/Akt pathway in gefitinib-sensitive NSCLC cell lines harboring wild-type and mutant EGFR (Engelman *et al*, 2005). Signalling through HER3 is fundamentally different from the other members of the HER family since HER3 has no tyrosine kinase activity but directly activates PI3K. Based on these data, we hypothesised that HER3 gene copy number could be relevant for gefitinib sensitivity, and increased copy number the HER3 gene could enhance gefitinib sensitivity in EGFR+ patients. This hypothesis was tested through the investigation of the HER3 status by fluorescence *in situ* hybridisation (FISH) in a cohort of NSCLC patients treated with gefitinib and previously evaluated for phospho-Akt (P-Akt) expression by immunohistochemistry (IHC), EGFR and HER2 status by FISH and DNA sequencing (Cappuzzo *et al*, 2004, 2005a, 2005b).

## METHODS

### Patient population

NSCLC patients included in this study were treated at three Italian institutions: Bellaria-Maggiore Hospital (Bologna), Scientific Institute University Hospital San Raffaele (Milano) and Policlinico Monteluce (Perugia). The cohort included 82 patients with advanced NSCLC accrued onto the AKT prospective clinical trial (Cappuzzo *et al*, 2004) or consecutively accrued from the Expanded Access Study (EAS) of gefitinib that followed the Akt trial. Eligibility for both studies included histologically confirmed NSCLC with measurable, locally advanced or metastatic disease, progressing or relapsing after chemotherapy, or medical contraindications for chemotherapy. Patients were classified as never smoker, former smoker (quit smoking more than 6 months before starting gefitinib therapy) or current smoker (quit smoking less than 6 months before starting gefitinib therapy or active smokers). The AKT study was approved by the Bellaria Hospital institutional ethical review board and written informed consent was obtained from each patient before enrollment. In the subgroup of EAS patients, the IRB approval was obtained according to Good Clinical Practice, and a specific written informed consent was obtained from each patient (EAS consent form, Italian version).

Patients received gefitinib (250 mg day^−1^) and were evaluated for response after 2 months according to the RECIST criteria (Therasse *et al*, 2000). Tumour response was assessed by computer tomography scan, with a confirmatory evaluation repeated in patients with complete response, partial response, and stable disease at least 4 weeks after the initial determination of response. Outcome in these patients was previously reported and associated with EGFR (Cappuzzo *et al*, 2005a) and HER2 status (Cappuzzo *et al*, 2005b). Among the patients'characteristics, female gender and never smoking status were significantly associated with better response; and female gender, adenocarcinoma and bronchioloalveolar histology and performance status 0–1 were significantly associated with longer survival.

No clinical or biological characteristics were used for patient selection for this study; the single criterion considered was the availability of tumour tissue and availability of information on P-Akt, EGFR and HER2 FISH status.

### Tissue preparation, FISH and IHC analyses

Sections from paraffin-embedded tissue blocks containing representative malignant cells obtained at time of diagnosis were used for this analysis. Histopathological classification was determined on hematoxylin–eosin (HE)-stained sections based on the World Health Organization criteria (Travis *et al*, 1999). The HE-stained slide was also used as a reference for selection of tumour foci to be analysed in the FISH assay. The HER3 FISH probe was prepared from the BAC clone RP11-603J24; acquired from BACPAC Resource Center (Children's Hospital Oakland Research Institute, Oakland, CA, USA). Single colony culture was used for amplification of the selected DNA sequences and the extracted BAC DNA was tested for the presence of HER3 sequences with PCR primers as follows: forward HER3 primer: 5′- GACATCAAGCATAATCGGCC-3′; reverse HER3 primer: 5′- CAGGACAAGCACTGACCAG-3′ and mapped by FISH to normal human karyotype. HER3 DNA was labeled by nick translation reaction with SpectrumRed-conjugated dUTP (Nick Translation Kit and reagents from Vysis/Abbott Laboratories) according to the manufacturer's instructions. The CEP12 (D12Z3) SpectrumGreen probe (Vysis/Abbott Lab, Downers Grove, IL, USA) was used as a control for chromosome 12 aneusomy.

Dual-target, dual-color FISH assays were performed as previously described (Cappuzzo *et al*, 2005a), using a combination of 120 ng HER3-SR, 5 ng CEP12-SG per hybridisation area. Briefly, sections were deparaffinised in CitriSolv (Fisher Scientific, Pittsburgh, PA, USA), dehydrated in 100% ethanol, incubated in 2 × saline sodium citrate buffer (2 × SSC; pH 7.0) at 75°C for 15–25 min, and digested with proteinase K (0.25 mg ml^−1^ in 2 × SSC; pH 7.0) at 37°C for 15–25 min. After dehydration in an ethanol series, the probe set was applied and slides were incubated at 80°C for 8–10 min for codenaturation of chromosomal and probe DNA, Hybridisation was allowed to occur at 37°C for approximately 40 h and posthybridisation washes were performed in 1.5 M urea and 0.1 × SSC (pH 7.0–7.5) at 45°C for 30 min and in 2 × SSC for 2 min at room temperature. After dehydration in graded ethanol series, 4′6-diamidino-2-phenylindole (0.30 mg ml^−1^ in Vectashield mounting medium; Vector Laboratories, Burlingame, CA, USA) was applied to the samples for chromatin counterstaining. Copy numbers of the HER3 gene and chromosome 12 centromere probes were assessed and recorded independently in at least 100 nonoverlapping nuclei from distinct tumour foci. Analysis was performed independently by two observers (LT, MVG) blinded to the patients' clinical characteristics and other receptors' status. According to the frequency of tumour cells with specific number of copies of the HER3 gene and chromosome 12 centromere, patients were classified into two strata: HER3 FISH negative (FISH−), with no or low genomic gain (⩽4 copies of the gene in >40% of cells) and HER3 FISH positive (FISH+), with high level of polysomy (⩾4 copies of the gene in ⩾40% of cells) or gene amplification (presence of tight gene clusters, a ratio gene/chromosome per cell ⩾2, or ⩾15 copies of the gene in ⩾10% of analysed cells).

### Statistical analysis

Differences between the FISH groups were compared by Fisher's exact test or *χ*^2^ test for qualitative variables and Student's by *t*-test. Normality of the distribution was assessed by Kolmogorov–Smirnov test. Time to progression (TTP), overall survival (OS) and the 95% confidence intervals were evaluated by the Kaplan–Meier method (Kaplan and Meier, 1985), comparing the FISH groups by log-rank test.

## RESULTS

### HER3 FISH

**Figure 1:**
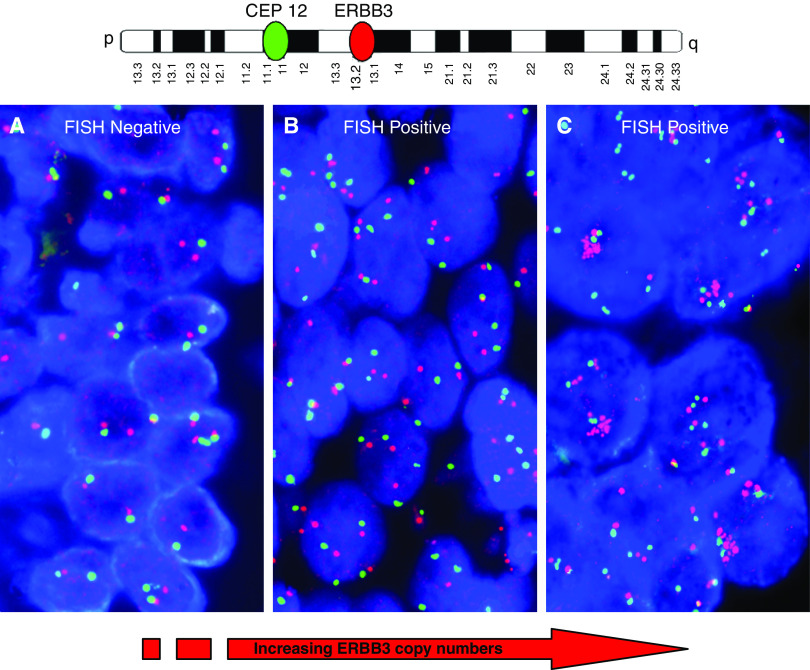
FISH with the HER3 probe. (**A**) No genomic gain (pattern: disomy); (**B**) high gene and chromosome copy numbers and (**C**) gene amplification.

Among 82 NSCLC patients, no or low genomic gain for the HER3 gene (HER3−) was found in 73.2% of cases, whereas high polysomy and gene amplification (HER3+) was detected in 26.8% (Figure 1). Among clinical characteristics (Table 1), HER3+ was significantly associated with female gender (*P*=0.02) and never smoking history (*P*=0.02), while no significant association was found with age, histology, stage and performance status.

**Figure 2:**
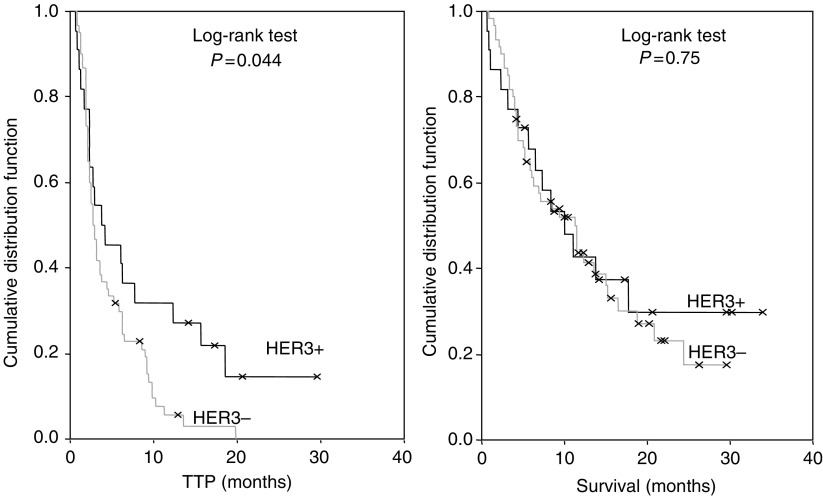
TTP and survival curves for patients with HER3+ and HER3− tumours. Median TTP and survival were 3.7 and 10.1 months in HER3+ and 2.7 and 11.3 months in HER3− (*P*=0.044 and 0.75, respectively).

Table 2 shows the association between HER3 FISH patterns and response to treatment, TTP and OS after treatment. No significant difference in overall response (OR, including complete and partial response), disease control rate (DCR, including OR and stable disease), TTP, and OS was observed between the two HER3 FISH strata. In the HER3+ group, OR was 18.2% and DCR was 45.5%, which was not significantly better than observed in the HER3− cohort (OR=11.7%, *P*=0.47; DCR=35%, *P*=0.38). HER3+ patients had a significantly longer TTP (3.7 *vs* 2.7 months, *P*=0.044), with no difference in terms of survival (10.1 *vs* 11.3 months, *P*=0.75, Figure 2).

### EGFR, HER2, P-AKT and HER3 association

#### Efficacy analysis

Epidermal growth factor receptor and HER2 gene copy number by FISH, mutations in the tyrosine kinase domain of the EGFR and HER2 genes by DNA sequencing and presence of phosphorylated AKT by immunohistochemistry have been previously determined in this cohort (Cappuzzo *et al*, 2005a, 2005b). Combining those results with the current analysis, it was verified that HER3 gene gain was observed more frequently in patients with EGFR gene gain (*P*=0.06), and HER2 gene gain (*P*=0.055), without a significant association with EGFR gene mutations (*P*=0.17) and Akt phosphorylation (*P*=0.47) (Table 3).

**Figure 3:**
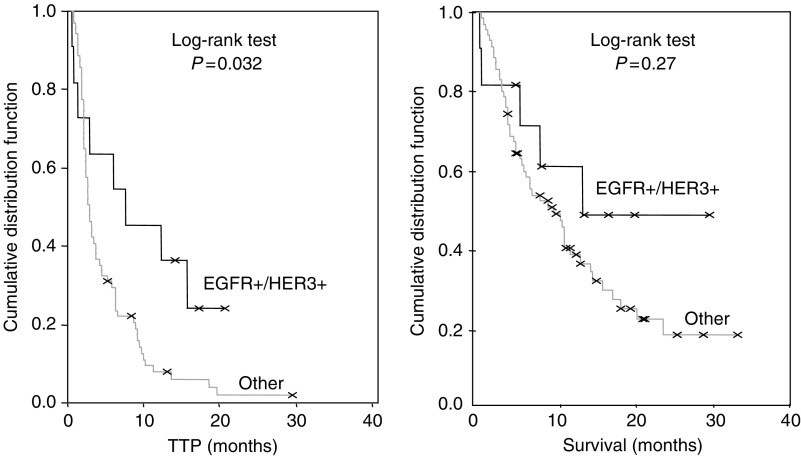
TTP and survival according to HER3/EGFR FISH status. TTP and survival were 7.7 and 13.8 months in EGFR+/HER3+ and 2.7 and 10.1 months in patients negative for HER3 and/or EGFR (*P*=0.032 and 0.27, respectively).

Table 4 shows the results in terms of OR and DCR in subsets of patient stratified according to EGFR FISH and HER3 status. Compared to patients with tumours negative for EGFR and/or HER3, individuals with tumours positive for both EGFR and HER3 (EGFR FISH+/HER3+) had a significantly higher OR (36.4 *vs* 9.9%, *P*=0.037) and a significantly longer TTP (7.7 *vs* 2.7 months, *P*=0.032), with a non significant trend toward longer survival (13.8 *vs* 10.1 months, *P*=0.27, Figure 3). Patients with tumours positive for both EGFR and HER3 had similar outcome than patients EGFR FISH+/HER3− (OR=36.4 *vs* 29.4%, *P*=1.0; DCR=63.6 *vs* 58.8%, *P*=1.0; TTP=7.7 *vs* 9.0 months, *P*=0.32, and OS=13.8 *vs* 18.7 months, *P*=0.85). In this study cohort, 13 patients (7 HER3− and 6 HER3+) had tumours with EGFR gene mutations. No difference was observed among EGFR mutation+ patients according to HER3 status and, because of the small number of patients in each group, outcome was not statistically compared. However, responders were observed in the EGFR mutation+/HER3+ group (three patients) and in the EGFR mutation+/HER3− group (five patients).

Nine patients had tumours positive for both HER2 and HER3. In this group of individuals, OR (33 *vs* 11%, *P*=0.063), DCR (56 *vs* 36%, *P*=0.28), TTP (7.7 *vs* 2.9 months, *P*=0.1) and OS (13.8 *vs* 10.9 months, *P*=0.63) were not significantly different from patients with tumours HER2− and/or HER3−. Among the nine HER2+/HER3+ patients, only three were also EGFR FISH− and they presented no response, the median TTP was 2.3 months, and the median OS was 2.4 months.

Tumours from a total of 16 patients were both HER3+ and P-Akt+. In this group of patients, OR (25 *vs* 11%, *P*=0.22), DCR (44 *vs* 37%, *P*=0.62), TTP (3.7 *vs* 2.9 months, *P*=0.20) and OS (8.3 *vs* 11.5 months, *P*=0.72) were not significantly different than observed in HER3− and/or P-Akt− individuals. Importantly, among the eight cases HER3+/P-Akt+ and EGFR FISH−, no objective response was detected, TTP was 2.4 months and median OS was 6.5 months. Conversely, among the eight cases HER3+/P-Akt+ and EGFR FISH+, four patients responded, two had disease stabilisation, and only two progressed.

## DISCUSSION

In the present study, we analysed the HER3 gene status by FISH in a cohort of NSCLC patients treated with gefitinib and observed that increased HER3 gene copy number had a low impact in drug sensitivity. Although TTP was significantly longer in HER3+ patients, response rate and survival of patients with increased HER3 gene copy number was not significantly different from the HER3− patients. The lack of a strong association with gefitinib sensitivity is not surprising because gefitinib is a TKI and HER3 lacks TK activity; thus, it can only signal in the context of a receptor heterodimer. Therefore, the best way to evaluate the impact of HER3 on gefitinib sensitivity is in combination with other EGFR family members, particularly EGFR and HER2.

Combining the EGFR and HER3 gene status determined by FISH, we observed that patients positive for both receptors (EGFR+/HER3+) had a significantly higher response and longer TTP, with a nonsignificant trend toward longer survival. Recent data indicate that NSCLCs responding to anti-EGFR therapy are likely to express HER3 at significantly elevated levels (Ono *et al*, 2004; Engelman *et al*, 2005). Moreover, preclinical data demonstrate that gefitinib inhibits the growth of HER2 overexpressing cells possibly by sequestration of HER2 and HER3 receptors in an inactive heterodimer configuration with EGFR (Anido *et al*, 2003). In the present study, although the better outcome was observed among HER3+/EGFR+ patients, the survival advantage was not statistically significant. Importantly, regardless of the method used for EGFR assessment (gene copy number by FISH or mutation analysis by DNA sequencing) no difference was observed among EGFR+ patients according to HER3 status. By contrast, our previous analysis conducted in the same cohort of patients demonstrated that HER2 gene increased copy number enhances gefitinib sensitivity in EGFR+ patients (Cappuzzo *et al*, 2005b), with a significant difference in outcome for patients EGFR+/HER2+ and EGFR+/HER2−. These findings suggest that HER3 is less relevant for gefitinib sensitivity than HER2.

Previous data have shown that the activation of the antiapoptotic PI3K-Akt pathway occurs in patients with mutation in the TK domain of EGFR (Sordella *et al*, 2004) or gene amplification (Cappuzzo *et al*, 2005a), and Akt activation results in increased gefitinib sensitivity (Cappuzzo *et al*, 2004). Because HER3 couples EGFR to the PI3K-Akt pathway in gefitinib-sensitive NSCLC cell lines harboring both wild-type and mutant EGFR (Engelman *et al*, 2005), we also investigated the correlation of HER3 with P-Akt. Increased HER3 gene copy number was not associated with P-Akt+ status, and the outcome of patients positive for both HER3 and P-Akt was not significantly different than patients negative for HER3 and/or P-Akt. Although the small number of patients precludes any firm conclusion, it is interesting to note that no response occurred among patients EGFR−/HER3+/P-Akt+, while a clear benefit was observed among EGFR+/HER3+/P-Akt+ patients. This finding is not surprising because sensitivity to gefitinib is not *per se* depending on HER3 or P-Akt status, but is related to the presence of the target EGFR, as demonstrated in preclinical (Engelman *et al*, 2005) and clinical models (Cappuzzo *et al*, 2005a, 2005b).

Why did this study fail to demonstrate a clinical advantage for HER3+ patients? The major difference with previous studies is that the present report is the first conducted in gefitinib-treated patients and not on cell lines (Anido *et al*, 2003; Sordella *et al*, 2004; Engelman *et al*, 2005). Another important difference is that this investigation was performed at the genomic rather than the protein level. Our previous findings regarding EGFR and HER2 (Cappuzzo *et al*, 2005a, 2005b) showed that protein expression was positively correlated with genomic status, but FISH results had a higher correlation with clinical outcome than immunohistochemistry results. However, it is conceivable that the same conclusion does not apply for HER3 and epigenetic, post-transcriptional and translational mechanisms could be responsible for an association between higher levels of expression and response to gefitinib. The scarce amount of tumour tissue remaining from our patients has precluded additional analyses to test other hypotheses. Finally, we cannot exclude that the present study was not enough powered to detect a potential benefit for patients with increased copy number of the HER3 gene.

In conclusion, our results suggest that increased copy number of the HER3 gene does not significantly enhance gefitinib sensitivity in EGFR+ NSCLC patients, therefore HER3 FISH is not likely to be useful for selection of NSCLC patients for TKI therapy. Further prospective studies in larger cohorts are warranted in order to confirm these findings.

## Figures and Tables

**Table 1:** Patient characteristics and HER3 FISH status in NSCLC

	**HER3 FISH status**			
**Characteristics**	**Total** ***N*=82** **No (%)**	**Positive** ***N*=22** **No (%)**	**Negative** ***N*=60** **No (%)**	***P***
*Sex*				
Male	57 (69.5)	11 (50.0)	46 (76.7)	
Female	25 (30.5)	11 (50.0)	14 (23.3)	0.02*
				
*Age (years)*				
Median	61.5	59.5	62	
<62	44 (53.7)	13 (59.1)	31 (51.7)	0.55
⩾62	38 (46.3)	9 (40.9)	29 (48.3)	
				
*Histology*				
Adenocarcinoma^a^	43 (52.4)	12 (54.5)	31 (51.7)	0.59
Bronchioloalveolar^a^	9 (11.0)	3 (13.6)	6 (10.0)	*P* (a*vs*^b^)
Squamous cell^b^	23 (28.0)	5 (22.7)	18 (30.0)	
Large cell^b^	1 (1.2)	1 (4.5)	0 (0)	
Undifferentiated^b^	6 (7.3)	1 (4.5)	5 (8.3)	
				
*Performance status*				
0	43 (52.4)	12 (54.5)	31 (51.7)	0.7
1	30 (36.6)	7 (31.8)	23 (38.3)	*P* (0+1 *vs* 2)
2	9 (11.0)	3 (13.6)	6 (10.0)	
				
*Smoking status*				
Never smoker	10 (12.2)	6 (27.3)	4 (6.7)	0.02*
Former smoker	30 (36.6)	6 (27.3)	24 (40.0)	*P* (Never *vs* others)
Current smoker	42 (52.2)	10 (45.5)	32 (53.3)	

* Statistically significant.

HER3 FISH− corresponds to no or low gain in copy numbers for the HER3 gene and HER3 FISH+ corresponds to high levels of gain for HER3 gene copy number (high polysomy and gene amplification).

a Grouping for statistics.

b Grouping for statistics.

**Table 2:** Objective response rate, disease control rate, TTP and survival analysis in NSCLC patients treated with gefitinib whose tumours showed no or low gain in copy numbers for the HER3 gene (HER3 FISH−) and high levels of gain for HER3 gene copy number (HER3 FISH+)

		**HER3 FISH status**		
**Outcome**	**Total Pts** ***N*=82** **No. (%)**	**Positive** ***N*=22** **No. (%)**	**Negative** ***N*=60** **No. (%)**	***P*-value**
Total	82 (100)	22 (26.8)	60 (73.2)	
Objective Response rate (OR)	11 (13.4)	4 (18.2)	7 (11.7)	0.47
Disease control rate (DCR=OR +stable disease)	31 (37.8)	10 (45.5)	21 (35.0)	0.38
Progressive Disease	51 (62.6)	12 (54.5)	39 (65.0)	
Time to progression (months)	2.9	3.7	2.7	0.044*
Median survival (months)	11	10.1	11.3	0.75

* Statistically significant.

**Table 3:** Association of HER3 FISH status and EGFR FISH, EGFR mutation, HER2 FISH and P-Akt status

		**HER3 FISH status**		
**Markers**	**Total Pts** ***N*=82** **No. (%)**	**Positive** ***N*=22** **No. (%)**	**Negative** ***N*=60** **No. (%)**	***P*-value**
EGFR FISH+^a^	28	11 (39.3)	17 (60.7)	0.06
EGFR FISH−a	54	11 (20.4)	43 (79.6)	
HER2 FISH+^b^	21	9 (42.9)	12 (57.1)	0.055
HER2 FISH−b	61	13 (21.3)	48 (78.7)	
EGFR Mutation+^a^	13	6 (46.2)	7 (53.8)	0.17
EGFR Mutation−a	60	15 (25.0)	45 (75.0)	
P-AKT+^a^	52	16 (30.8)	36 (69.2)	0.47
P-AKT−a	26	6 (23.1)	20 (76.9)	

a According to Cappuzzo *et al* (2005a).

b According to Cappuzzo *et al* (2005b).

**Table 4:** Outcome of NSCLC patients treated with gefitinib according to the combined status of EGFR FISH and HER3 FISH

**Markers**	**Total**	**OR (%)**	**SD+PD (%)**	**DCR (%)**	**PD (%)**	**TTP (months)**	**OS (months)**
EGFR+/HER3+^a^	11	4 (36.4)	7 (63.6)	7 (63.6)	4 (36.4)	7.7	13.8
EGFR+and/or HER3+^b^	39	9 (23.1)	30 (76.9)	20 (51.3)	19 (48.7)	5.9	13.8
EGFR+/HER3−c	17	5 (29.4)	12 (70.6)	10 (58.8)	7 (41.2)	9	18.7
EGFR− and/or HER3−d	71	7 (9.9)	64 (90.1)	24 (33.8)	47 (66.2)	2.7	10.1
EGFR−/HER3+^e^	11	0	11 (100)	3 (27.3)	8 (72.7)	2.7	7.3
EGFR−/HER3−f	43	2 (4.7)	41 (95.3)	11 (25.6)	32 (74.4)	2.6	8.5
*P (*a*vs*^c^)		1		1		0.32	0.85
*P (*a*vs*^d^)		0.037*		0.09		0.032*	0.27
*P(*a*vs*^e^)		0.09		0.19		0.32	0.24
*P (*b*vs*^f^)		0.014*		0.017*		0.002*	0.25

* Statistically significant.

a Groups compared statistically significant.

b Groups compared statistically significant.

c Groups compared statistically significant.

d Groups compared statistically significant.

e Groups compared statistically significant.

f Groups compared statistically significant.
